# Case Report: Clinical manifestation and dental management of Papillon-Lefèvre syndrome

**DOI:** 10.12688/f1000research.16042.1

**Published:** 2018-09-06

**Authors:** Yasmin Mohamed Yousry, Amr Ezzat Abd EL-Latif, Randa Youssef Abd El-Gawad

**Affiliations:** 1 Pediatric Dentistry and Dental Public Health, Faculty of Dentistry, Cairo University, Cairo, Egypt

**Keywords:** Papillon – Lefèvre syndrome, Periodontitis, Premature tooth loss, Palmoplantar keratosis.

## Abstract

**Background: **Papillon-Lefèvre syndrome (PLS) is considered a rare syndrome, which is characterized by the presence of palmar-plantar hyperkeratosis and aggressively progressing periodontitis that finally leads to premature loss of both deciduous and permanent teeth.

**Case report:** A four-year-old Egyptian boy presented with a maternal complaint that her child suffers from early loss of many teeth, presence of loose teeth along with an asymptomatic swelling related to the upper anterior area. The patient was diagnosed with PLS. A symptomatic management and prevention program was followed and the swelling was excised; afterwards diagnosed as peripheral ossifying fibroma.

**Conclusion:** Early recognition and intervention for patients with PLS is essential to avoid the threat of being edentulous if left unmanaged.

## Introduction

Papillon-Lefèvre syndrome (PLS) is an autosomal recessive disorder that typically becomes apparent from one to five years of age, which coincides with the timing of eruption of primary dentition. The estimated prevalence of the syndrome is 1–4 cases per million individuals ^1^.

The exact etiopathogenesis of the syndrome is relatively unclear and different etiological factors have been suggested, such as immunologic, genetic or bacterial, but recently it was suggested that mutations of cathepsin C gene, which results in deficiency of cathepsin C enzymatic activity, to be the possible etiological factor. This was supported by the fact that expression of the cathepsin C gene occurs mainly in epithelial regions, such as the soles, palms and keratinized oral gingiva, which are the most affected areas in patients with PLS ^2^.

An important feature of the syndrome is the presence of palmoplantar hyperkeratosis; its onset usually occurs between the ages of one to four years and usually involves the palms of the hands and soles of the feet ^3^. Another major feature is severe gingivostomatitis and periodontitis. Deciduous teeth usually erupt in normal sequence, timing and with normal structure and form, although it was reported that some cases may have microdontia and incomplete root formation ^4^.

First, the gingiva becomes inflamed and then rapid destruction of periodontium occurs. This is manifested in the form of redness and swelling in the gingiva with severe bone resorption and periodontal pockets. Patients usually suffer from looseness, drifting, migration, and exfoliation of teeth so that by the age of 4–5 years all primary teeth are prematurely exfoliated and the same cycle is repeated with permanent teeth ^5^.

A multidisciplinary approach for the management of cases with PLS is usually required and periodontal treatment, if started early, will decrease the rate of periodontal destruction ^6^.

We hereby report a rare case that, to the best of our knowledge, may be the first for a child with PLS together with peripheral ossifying fibroma lesion that is not a characteristic feature for the syndrome.

## Case report

A four-year-old Egyptian boy presented to the Pediatric Dental Clinic, Faculty of Dentistry, Cairo University, suffering from premature loss of anterior teeth, friable and bleeding gums and swelling related to the upper anterior region. Medical history revealed absence of any medical problems; family history revealed that neither parents nor siblings had the same problem and the parents were not of consanguineous marriage.

**Figure 1.**
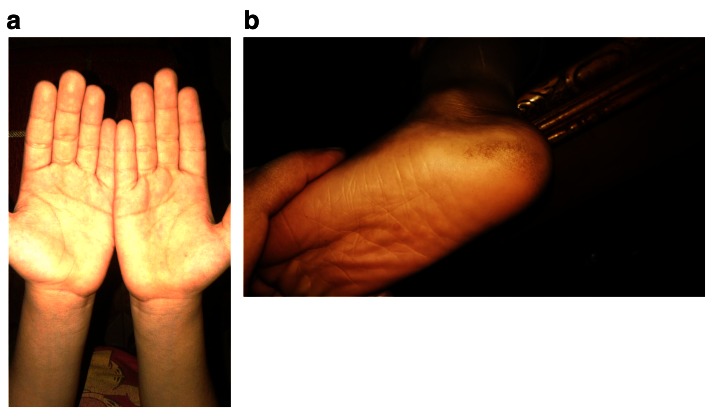
Photographs of ( **a**) the palms of the hands showing normal skin and ( **b**) the soles of the feet showing very slight hyperkeratosis.

**Figure 2.**
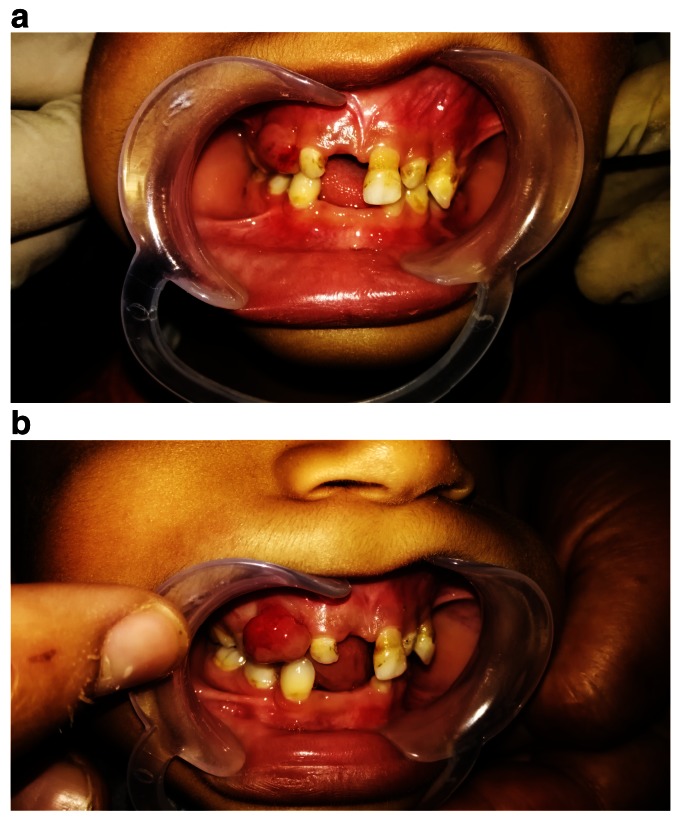
Intraoral photographs showing ( **a**) severe gingival recession and inflammation, especially in anterior region, and aggressive periodontitis; ( **b**) swelling related to the maxillary right missed canine region extending toward occlusal surface.

Examination of the palms of the hand revealed normal skin, while the soles of the feet revealed very slight hyperkeratosis ( Figure 1a,b). Intraoral examination revealed severe gingival recession; inflammation especially in anterior region; aggressive periodontitis; mobility of maxillary left central incisor and canine, with swelling related to the maxillary right missed canine region extending toward occlusal surface. The swelling appeared as a solitary rounded lesion, with onset gradual for 2 months. The size of the swelling was 4×4 mm, and upon palpation it was not tender but slightly hemorrhagic ( Figure 2a,b).

**Figure 3.**
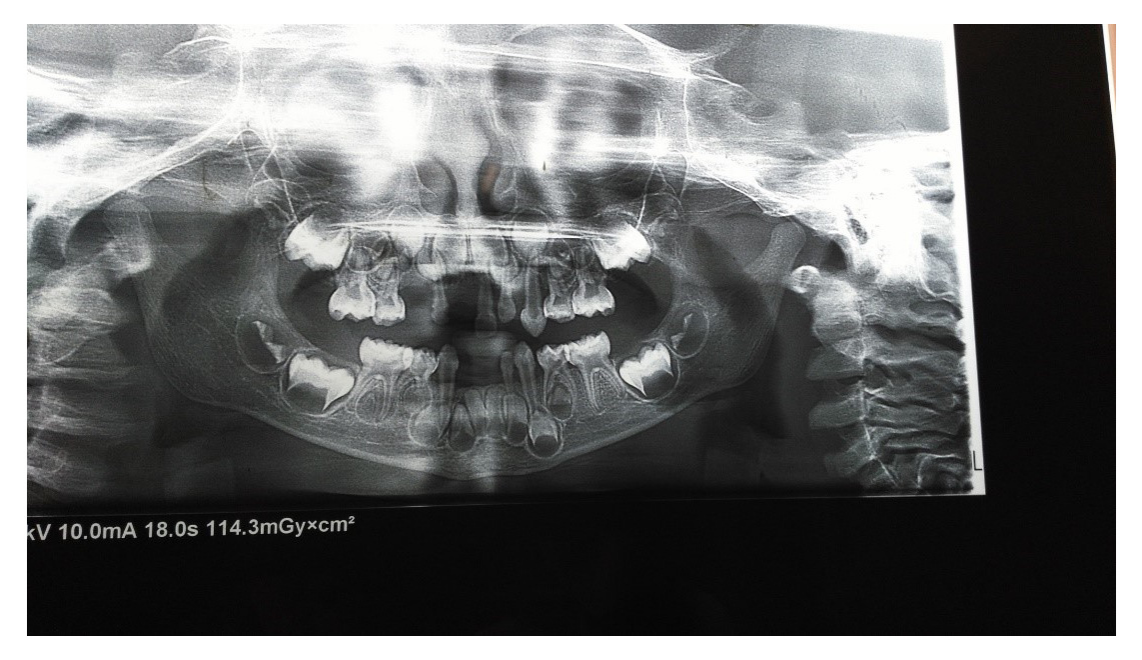
Panoramic radiograph showing severe destruction and loss of alveolar bone in both maxillary and mandibular arch, especially in the anterior region and anterior teeth appear as if floating in air without surrounding alveolar bone.

Radiographic examination showed severe destruction and loss of alveolar bone ( Figure 3). Lab investigations were normal ( Table 1).

**Table 1.** Lab investigations results showing that serum calcium and phosphorus level is normal, Alkaline phosphatase level is normal and the complete blood work is normal.

Test name	Results	Units	Reference range
HB &indeses			
Haemoglobin	11.6	gm/dl	11.5 - 16.0
Red cell count	4.23	mil/cmm	4.0 – 5.6
Haematocrit (pcv)	36	%	36- 46
Red blood cell indices			
MCV	78	fL	77-95
MCH	27	pg	25 - 30
MCHC	30	%	30- 34
TLC & Differential			
White cell count	10.200	Thousand/cmm	4.0-13
Basophils	0	/cmm	0- 2
Eosinophils	1	/cmm	1 - 4
Staff	2	/cmm	0- 6
Segmented	40	/cmm	37- 75
Lymphocytes	50	/cmm	20-45
Monocytes	7	/cmm	2-10
PLT			
Platelets count	171	Thousand/cmm	150- 450
MPV	7.2	fL	6.5- 12
PDW	15.6	%	9 - 17
PCT	0.21	%	0.1 -0.5
P-LCR	14	%	13- 43
Clinical chemistry report			
ALP,serum	233	U/ l	( up to 640 )
Calcium (total) ,Serum	9.8	mg/dl	(8.6- 10.2)
Phosphorous	4.8	mg/dl	( 4.0- 7.0 )
Liver function tests			
Alkaline phosphatase	534	U/L	180- 1200

Taking into consideration the clinical features and investigations, a diagnosis of PLS was confirmed.

### Dental management of the case

Conventional periodontal treatment in the form of scaling and root planning was performed. Antibiotic amoxicillin and metronidazole (250 mg, 3 times daily) for one week along with a mouth rinse (0.2% chlorhexidine gluconate, 10 mL twice daily) was prescribed to the patient ^7^.

Extraction of the maxillary left central and canine teeth was advised, but the parent refused even after the risk was explained of not extracting these loose teeth. 

**Figure 4.**
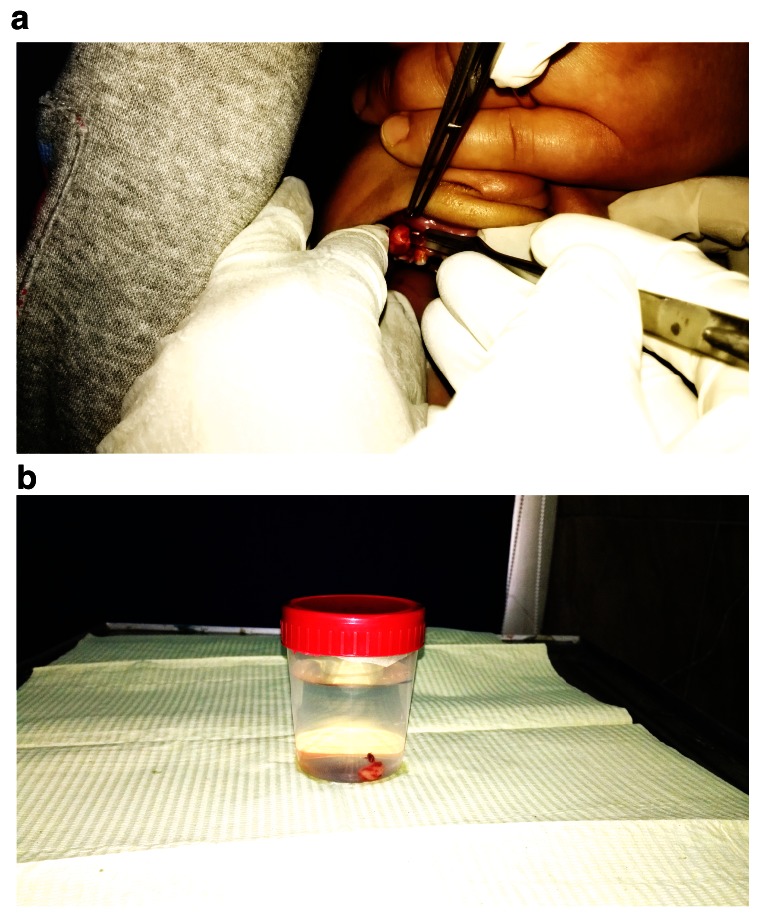
Photograph showing ( **a**) removal of the swelling and ( **b**) excisional biopsy of the swelling.

**Figure 5.**
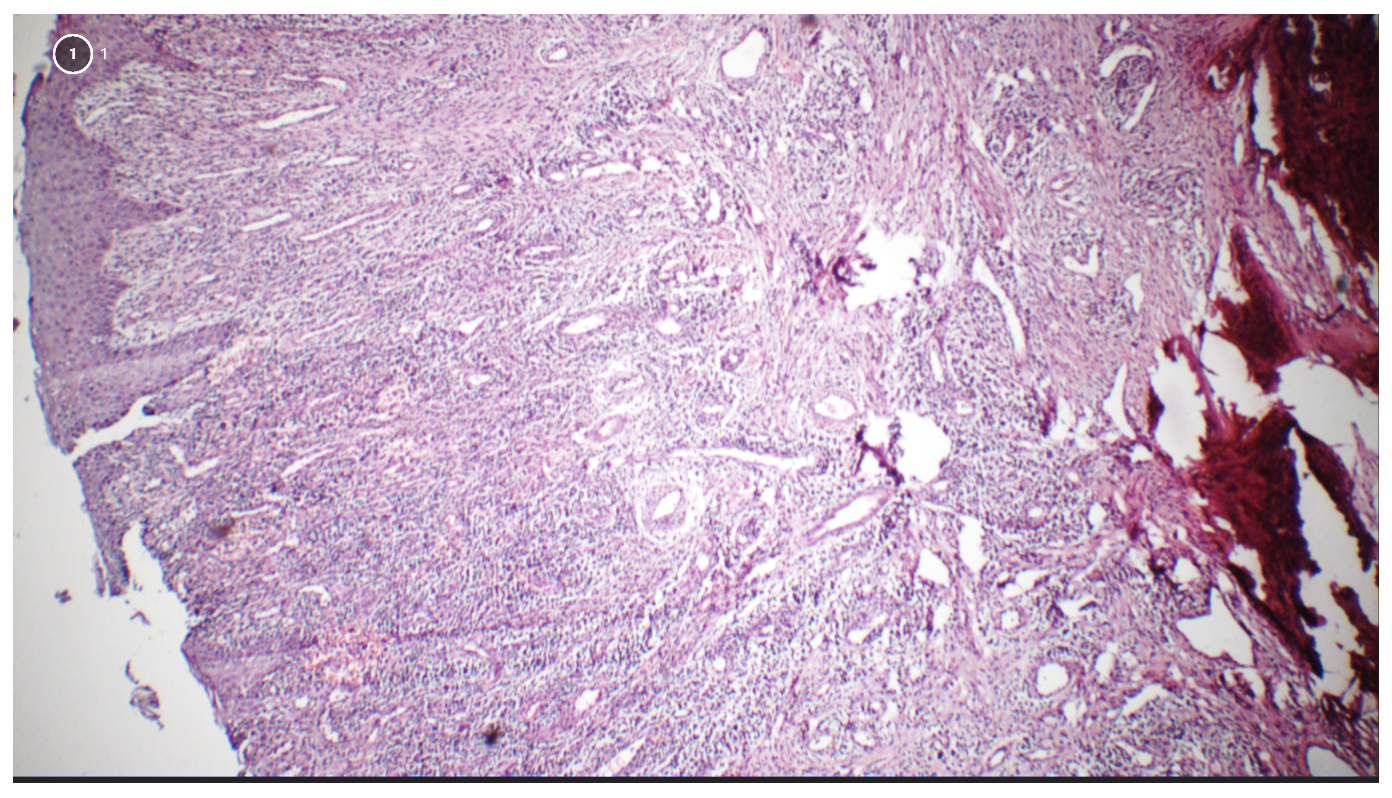
Histopathological image showing evidence of calcifications in the hypercellular fibroblastic stroma confirming the lesion as peripheral ossifying fibroma.

After laboratory investigations, excisional biopsy of the swelling was done under antibiotic coverage and local anesthesia. Thorough curettage of the adjacent periodontal ligament and periosteum was carried out to prevent recurrence ( Figure 4 a,b). Histopathological examination revealed the lesion as peripheral ossifying fibroma ( Figure 5).

The patient was educated for oral hygiene and scheduled for a follow-up visit every month for scaling and checking the condition of the patient.

**Figure 6.**
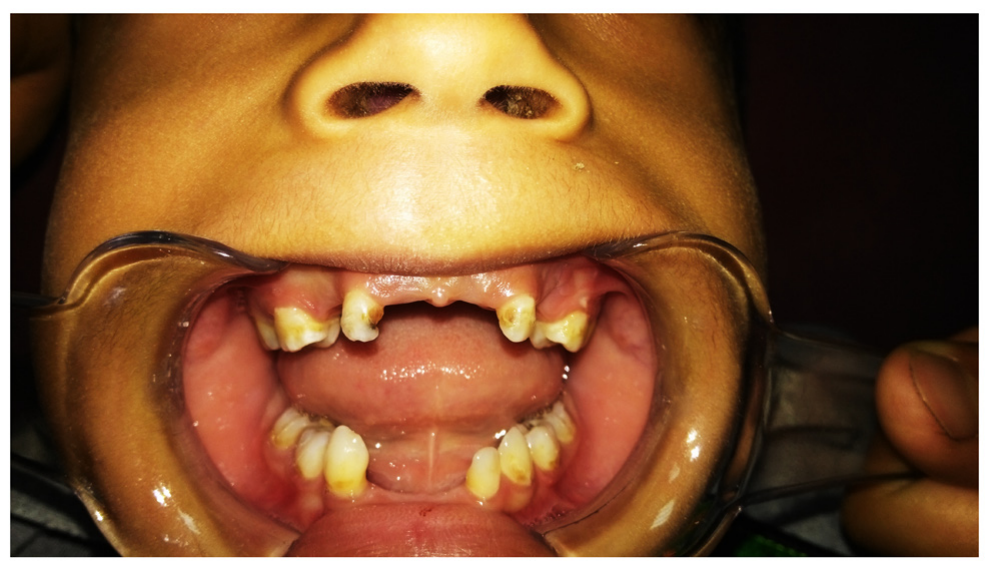
Follow-up photograph after 2 years showing loss of more teeth with no recurrence of the lesion.

**Figure 7.**
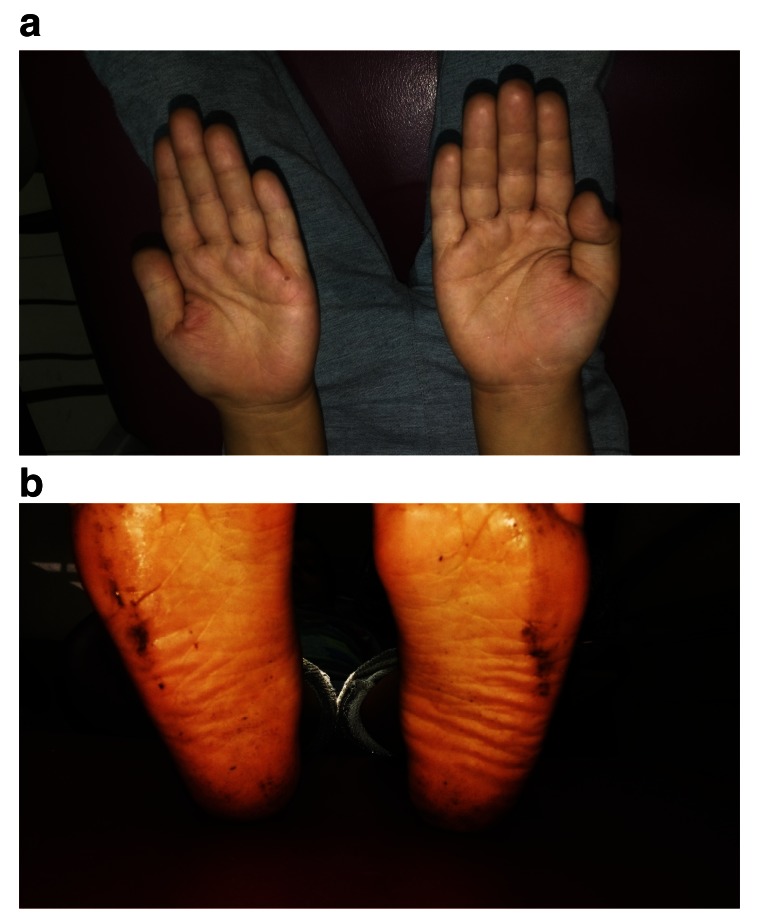
Follow-up photographs after 2 years showing ( **a**) absence of change in the palms of the feet and ( **b**) slight increase in keratosis in the soles of the feet.

The patient was followed up for 2 years during which loss of maxillary left central incisor occurred and extraction of loose upper left canine was done with no recurrence of the lesion ( Figure 6). The palms of the hands revealed no change, while examination of the soles of the feet showed slight increase in keratosis ( Figure 7 a,b).

## Discussion

Papillon-Lefèvre syndrome (PLS) is inherited as an autosomal recessive disorder where the parents of the patient with PLS should have the autosomal gene for the syndrome in order to manifest in their offspring. However, in the present case the parents are clinically healthy with no family history of the disorder. Studies have shown that when carrier parents for the affected gene mate, there is a 25% chance that they have an affected offspring ^8^. This could explain the reason that the child had the syndrome although his parents were clinically healthy.

The intraoral appearance of severe aggressive periodontitis, which appears at the age of 3–4 years following complete eruption of primary teeth as seen in this case, concurred with observations in similar reported cases in the literature where primary teeth develop normally but eruption is accompanied with severe gingivitis followed by periodontal destruction, resulting in early loss of primary teeth ^9^.

Ullbro *et al.* ^10^ suggested that the two major components of PLS (palmar-plantar hyperkeratosis and aggressively progressing periodontitis) are not related to each other, as these authors found absence of association between the degree of hyperkeratosis and severity of periodontitis. This is in accordance with our case as the degree of hyperkeratosis is slight although periodontitis is severe.

Acrodynia, hypophosphatasia and cyclic neutropenia are differential diagnoses of PLS. This case is not acrodynia due to absence of erythrocyanosis, insomnia, and teeth erupting prematurely with dystrophic enamel. It is not hypophosphatasia due to normal level of alkaline phosphatase and it is not cyclic neutropenia, as in cyclic neutropenia the palmoplantar hyperkeratosis is absent ^11^.

Management of cases with PLS should be multidisciplinary with dentists, dermatologists and pediatricians. Early diagnosis and management of oral problems help in reducing the undesirable sequelae of the syndrome. Following the treatment protocol for periodontal therapy proposed by Ullbro *et al.* ^10^ periodontal deterioration can be minimized. This includes: scaling and polishing; giving systemic antibiotics aimed at eliminating the reservoir of causative organisms; extraction of teeth having poor prognosis; giving instructions for maintenance of oral hygiene; and continuous monitoring and frequent recall appointments.

In the present case an early diagnosis of PLS and a treatment protocol minimized the periodontal deterioration and prevented further loss of other teeth. The parents were satisfied by these results. 

## Consent

Written informed consent for publication of the clinical details and images was obtained from the patient's mother.

## Data availability

All data underlying the results are available as part of the article and no additional source data are required.
