# Supplementary material for: Case Report: Clinical manifestation and dental management of Papillon-Lefèvre syndrome
Source: F1000Res. 2018 Sep 6;7:1420. [Version 1] doi: 10.12688/f1000research.16042.1 (PMC6178905; doi:10.12688/f1000research.16042.1)
Supplement: Peer review history

# Peer review: Referee response for version 1

**DOI:** 10.5256/f1000research.17520.r38617

**Published:** 2018-10-05

**Authors:** Noha Ezzat Sabet

**Affiliations:** 1 Department of Orthodontics, Faculty of Dentistry, Ain Shams University, Cairo, Egypt

The case report is quite informative, well written. Clearly and easily understood. The subject is addressed obviously and the diagnostic procedures are to clarify the point of interest. The results are professionally discussed and the conclusion that calls for early diagnosis to minimize the progress of dental loss and periodontal deterioration is of great interest. I think the authors should have clarified did they or did not restore the missing teeth. Also I would recommend a longer time of follow up to the case to ensure the condition of the permanent teeth after their eruption and to assure that its eruption time is not affected by the periodontal condition.

I have read this submission. I believe that I have an appropriate level of expertise to confirm that it is of an acceptable scientific standard.

# Peer review: Referee response for version 1

**DOI:** 10.5256/f1000research.17520.r38401

**Published:** 2018-09-19

**Authors:** Marwa Mokbel ElShafei

**Affiliations:** 1 Faculty of Dentistry, Misr International University, Cairo, Egypt

The case report is concerned about a case of a 4 year-old boy suffering from looseness of some teeth and loss of many others. Palmar-plantar hyperkeratosis is noticed on his palms and soles, although not severe but well detected. Aggressive progressive periodontitis is diagnosed as the cause of loss of teeth.

 A painless swelling is found on the gingiva related to the upper anterior teeth; this swelling was excised and diagnosed as a peripheral ossifying fibroma.  

 Follow up and scheduled scaling and polishing to prevent sequel of aggressive periodontitis is the management chosen for this patient.

- Another key word to be added "peripheral ossifying fibroma" 
- Another photomicrograph needed to confirm presence of calcification and a possible immunohistochemical staining with cathepsin C and with calcitonin is an option.
- State how long did the monthly follow up remained.
- How did you restore the lost permanent central incisor and how would you prevent future loss and looseness of teeth due to the syndrome's periodontitis. 

I have read this submission. I believe that I have an appropriate level of expertise to confirm that it is of an acceptable scientific standard.
